# Precision Vaccinology Approaches for the Development of Adjuvanted Vaccines Targeted to Distinct Vulnerable Populations

**DOI:** 10.3390/pharmaceutics15061766

**Published:** 2023-06-19

**Authors:** Branden Lee, Etsuro Nanishi, Ofer Levy, David J. Dowling

**Affiliations:** 1 Precision Vaccines Program, Boston Children’s Hospital, Boston, MA 02115, USA; branden.lee@childrens.harvard.edu (B.L.); etsuro.nanishi@childrens.harvard.edu (E.N.); ofer.levy@childrens.harvard.edu (O.L.); 2 Department of Pediatrics, Harvard Medical School, Boston, MA 02115, USA; 3 Broad Institute of MIT and Harvard, Cambridge, MA 02142, USA

**Keywords:** adjuvant systems, immune potentiators, immunomodulators, toll-like receptor (TLR) agonists, emulsions, aluminum salts, nanoparticles, polymers

## Abstract

Infection persists as one of the leading global causes of morbidity and mortality, with particular burden at the extremes of age and in populations who are immunocompromised or suffer chronic co-morbid diseases. By focusing discovery and innovation efforts to better understand the phenotypic and mechanistic differences in the immune systems of diverse vulnerable populations, emerging research in precision vaccine discovery and development has explored how to optimize immunizations across the lifespan. Here, we focus on two key elements of precision vaccinology, as applied to epidemic/pandemic response and preparedness, including (a) selecting robust combinations of adjuvants and antigens, and (b) coupling these platforms with appropriate formulation systems. In this context, several considerations exist, including the intended goals of immunization (e.g., achieving immunogenicity versus lessening transmission), reducing the likelihood of adverse reactogenicity, and optimizing the route of administration. Each of these considerations is accompanied by several key challenges. On-going innovation in precision vaccinology will expand and target the arsenal of vaccine components for protection of vulnerable populations.

## 1. Introduction

For several decades beginning in the 1920s, insoluble aluminum salts were the first and only adjuvants used to enhance vaccine efficacy against infectious diseases [1]. Since that period, several additional adjuvants have been included in approved vaccines (Table 1), from the oil-in-water (OIW) emulsion MF59 to the GSK adjuvant systems (AS). These developments signify a nearly-100-year timeline of interconnected developments in the fields of vaccinology and immunology [1,2].

Recently, the notable widespread employment of mRNA, viral vector, and adjuvanted vaccine technologies have had a profound global impact on reducing the impact of the COVID-19 pandemic. An estimated 10+ million deaths were prevented globally due to SARS-CoV-2 vaccinations, with a significant contribution attributed to the speed of pathogen-specific mRNA vaccine development [3]. However, while this innovation signifies a breakthrough in vaccinology and modern medicine, it also highlights an important challenge in the field. Different age groups presented with distinct responses to the mRNA vaccines. In particular, the mRNA vaccines demonstrate waning effectiveness, diminished protection, and heterogeneous efficacy, a phenomenon that is not restricted to but is especially pronounced in older adults [4,5,6,7]. This is likely a result of several factors, which include the variability in the magnitude and persistence of antigen expression and subsequent clearance from the encoded mRNA [8,9,10]. Ultimately, these heterogeneous responses invite further research into the biodistribution and pharmacometrics of this novel vaccine platform as gene therapies begin to be integrated into vaccinology. Nevertheless, a diminished protection for older adults is also seen in both non-mRNA vaccines against SARS-CoV-2 [11] and seasonal influenza vaccines [12,13]. A number of factors appear to be driving this differential response, from age-specific response to adjuvants [14,15], immune senescence, to potential diminishing immunization efficacy over time as a result of immune imprinting [16,17,18]. Considering these factors to inform rationally designed vaccines that effectively protect immunocompromised individuals and/or older adults remains an active area for improvement in current vaccine development streams.

A related paradigm also exists for immunizing younger populations. As the immune system undergoes dramatic changes in the early years of life, significant phenotypic differences emerge between pediatric and adult immune responses [19,20,21]. These differences manifest in a higher pediatric susceptibility and vulnerability to certain infectious diseases [22,23,24]. In fact, infectious diseases are amongst the leading global causes of pediatric mortality [25], which is at least partially due to their distinct immune responses. Further, the more distinct immunological phenotypes of young children as well the impact of transplacental maternal immunity contribute to significant differences in vaccine response in early life [26,27,28]. Thus, an important objective of modern vaccinology is defining safe approaches to shape immune responses in early life [29,30,31,32].

Similarly, individuals with weakened immune systems, such as those living with metabolic disorders and diabetes, immunocompromised populations, and people living with chronic viral infection and/or cancer, experience heightened susceptibility to infectious diseases [33] and suboptimal responses to immunizations [34,35]. While immunocompromised status represents a large and heterogenous population with diverse phenotypes and severity, there is an unmet need to enhance vaccine efficacy for this diverse group [36]. Defining immune pathways to trigger protective immunity in these populations, including optimal adjuvants, may be key to protection of these groups.

Overall, our growing understanding of different populations that vary by age, sex, and disease status coupled with the growing range of biotechnologies accessible to vaccinologists provide new opportunities to advance the field. Here, we discuss how these two streams are being integrated under the paradigm of precision vaccinology to enhance vaccine efficacy for distinct vulnerable populations. In this article, we focus primarily on applying these principles to small molecule-based adjuvant systems, the innate immune pathways they activate, as well as approaches to their formulation and delivery.

## 2. Precision Vaccinology Principles in Adjuvant Innovation, Discovery, and Development

### 2.1. Emerging Tools for Adjuvant Discovery

High-throughput screening (HTS) of small molecules has been leveraged by pharmaceutical and academic centers to expand our scientific toolbox. HTS has contributed to ~20–30% of late-stage clinical development candidates at several prominent pharmaceutical companies [37]. However, the same analyses suggest that ~60% of early-stage candidates discovered via HTS fail to reach later-stage applications. Several approaches have been employed to overcome this modest yield, including (1) developing more curated and druggable chemical libraries to optimize the source of testable drug candidates [38] and, (2) optimizing the screening platform itself—finding more applicable or translational in vitro systems to screen the chemical libraries. From engineered constructs for target-based screening to broad phenotypic screens for functional discovery, there has been a significant expansion in available tools to screen compounds. Some notable developments include the application of organ-on-chip technologies as screening tools and incorporation of microfluidic screening to enable ultra-high-throughput screening [39,40]. Extending to vaccine adjuvant discovery, innovation in phenotypic and targeted screens have identified multiple novel adjuvants [41,42,43].

The expanded menu of adjuvant discovery tools can advance precision vaccinology. In regard to the growth in target-based screening technologies, targeted screens can be steered towards addressing gaps in vaccine efficacy for distinct vulnerable populations. For example, infant populations often skew towards Th2-like immune responses and fail to mount sufficient Th1 responses [44,45]. This Th2-skewed response leaves pediatric populations at higher susceptibility and vulnerability to many serious complications arising from intracellular pathogens, such as viral respiratory infections [46,47]. Of note, activation of endosomal TLRs, such as TLR7 and/or -8, can shape the infant immune response towards a more adult-like Th1 response [48,49], which can enable effective protection against many respiratory viruses. Thus, with more advanced targeting screening tools for these endosomal targets, the arsenal of precision adjuvants available to vaccinate this vulnerable population can substantially advance. This framework can be more broadly applied to various vulnerable populations as we characterize population-specific immunity [50,51,52].

The expansion of phenotypic screening tools can enhance translation to advance precision-adjuvant discovery. With human in vitro models being developed for HTS, it is now possible to screen for molecules with optimal activity towards distinct populations [53]. Indeed, human primary immune cells have been directly used to screen for adjuvant discovery [54]. Using human primary cells from individuals from distinct populations (e.g., by age, sex, and/or co-morbidity), may enable discovery and development of bespoke adjuvants for vulnerable populations.

### 2.2. Innovations through Advanced Adjuvant Compositions

One of the largest bottlenecks in advancing novel lead candidates from discovery to clinical consideration is optimizing the function of the compounds via medicinal chemistry. Altering compound structure can improve on-target efficacy of compounds in addition to their pharmacodynamic and pharmacokinetic properties [55,56,57]. These types of medicinal chemistry approaches can correspondingly be applied to develop more ideal adjuvant compositions [58,59]. Evolving objectives for these optimized compounds include improving potency, efficacy, safety, and biological clearance. Additionally, an emerging focus of adjuvant medicinal chemistry involves ligating chemical bridges onto novel adjuvants to optimize integration into antigen formulation [60,61]. Thus, characterizing the active moiety of a molecule and adding bridges, such as phosphonate groups for alum absorption, that do not interfere with this moiety can be a powerful tool for translation/application. This impact is heightened with emerging drug-delivery technologies that enable targeting of and integration for specific tissues and even cells [62,63,64]. Thus, the inherent functionality of novel small-molecule adjuvants can be optimized and they can be accommodated into sophisticated delivery technologies.

In addition to improving the functionality of compounds, another important focus of medicinal chemistry effort for novel adjuvants is to remove any off-target effects, sometimes referred to as “wasted immunity”. Many discovered leads are compounds with pleiotropic activities. This can be the basis of drug-repurposing efforts that have come to define the growing field of polypharmacology. More often, however, this pleiotropic nature, which is notably linked to larger and unrefined biologics [65,66,67], accompanies adverse side-effects and safety concerns that contribute to the high attrition rates seen in the drug-development pipeline [68]. Indeed, several early- and late-stage vaccine adjuvant candidates, such as water in oil/squalene or poly(I:C), demonstrate potential adverse reactogenicity [69]. Potential reactogenicity can be addressed by reengineering biologic or chemically altering small molecule candidates to yield more precise functionalities [70,71]. Indeed, a precision medicine approach is key for discovery and development of safe and effective vaccines for distinct populations that vary in susceptibility to vaccine-associated adverse events [71].

## 3. Exploration of Immunization Sites to Unleash Maximal Effects in Target Populations

While significant research has been focused on expanding the tools available to formulate a vaccine, equally important is defining the immunogenicity needed to achieve protection [72]. To this end, all licensed vaccines have targeted routes of administration that maintain a localized and controlled antigen presentation, thereby avoiding the potential dangers of a systemic vaccine and the added potential benefit that some vaccines provide via depot effects [73,74,75]. Depending on route of administration, vaccines can unleash unique benefits reflecting distinct immunity mounted by different target tissues.

### 3.1. Intradermal, Subcutaneuous, Intramuscular—A Layered Lesson in Precision Vaccinology

The layers of tissue from skin to muscle provide several targets for immunization. Considering the different immune cell compositions found at each tissue layer, there are characteristic differences in immunity induced between intradermal (ID), subcutaneous (SC), and intramuscular (IM) vaccination [76].

ID injections enable preferential access to dermal dendritic cells (DCs) and Langerhans cells (LCs). Targeting these dermal sentinels facilitates highly-effective antigen presentation to skin resident and lymph-node-resident memory T cells via migration to lymphoid organs [77,78]. Furthermore, with >20 times the myeloid DCs and double the T cells spanning the human dermis compared to circulating blood [79], ID injections are an attractive approach to optimize vaccine immunogenicity. In fact, at an equivalent antigen dose, ID vaccination often induces greater immunogenicity than IM or SC vaccination [80]. Further, LCs are maintained as a steady-state cellular population, rendering them a highly promising target for precision vaccines for both older and younger populations [81,82]. However, the drawback of this route of administration is the inherent difficulty of physically injecting at the proper depth, further compounded by the heterogeneity of the dermal thickness across age groups, body types, and demographics [83,84,85,86]. As innovations move the field from the canonical needle-and-syringe approach to standardizable novel systems, such as dermal patches or laser-guided injection systems that can precisely deliver vaccine components, ID immunizations become an even more appealing option in precision vaccinology [87,88,89].

SC vaccinations, while historically considered a safe and efficacious route of administration, have been questioned of late. Concerns about safety and poor mobilization and ultimate presentation of antigen have highlighted some shortcomings of this route [90,91,92]. Further, new lines of research indicate that other routes may be more efficacious. Using the recent monkeypox public health emergency as a case-study, the Vaccinia Ankara vaccine was deployed for vulnerable populations. Historically, this vaccine was given with a two-dose regimen via SC immunization. However, it was retooled as a single dose ID immunization at 1/5th of the antigen dose and still maintained comparable protection [93,94,95,96].

IM injections remain the most common way to deliver vaccines. With a lower density of pain fibers compared to ID or SC layers and ample blood flow, IM is an ideal candidate for many types of formulations [97,98]. While the IM route may not proffer the same density of immune cells that ID layers do, the resiliency of muscle may enable a higher threshold of tolerance for a range of vaccine formulations.

### 3.2. Nose to Gut—Mucosal Immunity

While the IM, SC, and ID routes of vaccine delivery are effective for inducing systemic immunity to prevent severe illness, they may be suboptimal in preventing infection at the site of invasion. These considerations highlight a significant area for improvement in current vaccine development efforts, which has become growingly visible due to the ongoing COVID-19 pandemic: mucosal immunity [72,99,100]. Mucosal layers cover much of our respiratory, gastroenterological, and genital tracts and serve as a barrier against respiratory, enteric, and genital infections, respectively. Establishing mucosal vaccination approaches that can potentiate protection at this layer and can prevent infection from occurring in the first place is a key area for research and translation. From a public-health framework, developing a mucosal vaccine can be a future tool to better curb transmission of highly virulent strains and even eradicate the presence of malignant viruses. Pertinently, this is an even higher priority precision-vaccine objective, when routinely protecting vulnerable populations via this approach is still mostly technocritical.

There have been many modern advancements in mucosal vaccines, in both oral- and nasal-delivery systems. There have been several mucosal vaccine candidates with exciting preclinical results [101,102] and even some promising indications in clinical trials [103,104,105]. However, most of the candidates have mixed to moderate levels of protection, with observed variability across different age groups [106,107,108]. As a result, there are less than 10 licensed enteric vaccines and less than five licensed nasal vaccines, which includes two recently approved intranasal candidates against SARS-CoV-2 [109,110].

One of the major challenges in developing mucosal vaccines, which may explain the limited number of approved candidates, is ensuring a sufficiently immunogenic and durable response. Considering that the mucosal layer constitutes unique subsets of immune sentinels and a distinct mechanism of protection [111], growing research is focused on establishing important targets to stimulate in this context. Novel adjuvanted approaches have been described that may be unique or optimal to mucosal immunizations and as we better understand the correlates of protection and mucosal triggers, the efficacy of these vaccines will substantially improve [112]. Similarly, vaccine immunogenicity, especially that of mucosal vaccines, is effected by our microbiome in complex ways [113,114,115]. Characterizing how the microbiome impacts vaccine immunogenicity, may inform precision vaccination approaches. Overall, developing effective mucosal adjuvants may enable overcoming generally poor immunogenicity and heterogeneity of mucosal vaccines across different age groups.

However, adjuvanted approaches for mucosal vaccines, especially via a nasal route, need to be accompanied with a particular emphasis on safety. Intranasal influenza vaccine with *Escherichia coli* heat-labile toxin as a mucosal adjuvant has been associated with higher risk of developing Bell’s palsy [116]. While the association may be specific to this adjuvant, not with the specific route of administration, with the evidence that Bell’s palsy is also associated with parental SARS-CoV-2 mRNA vaccine administration [117], it is of heightened concern in intranasal contexts, considering its proximity to cranial nerves and the larger central nervous system.

With effective mucosal vaccines, we can strongly reduce the incidence and spread of pathogens, while protecting individuals before infection can manifest as disease. This can directly protect both infant and aged populations, which are more susceptible to multiple types of respiratory infections. Additionally, these vaccines can help overcome the durability and consistency challenges present for intramuscular vaccinations in these groups. Additionally, these vaccines can more directly target enteric diseases, a particular threat to global pediatric populations [25,118]. Thus, discovery and development of mucosal vaccines with optimized functionality remains an important aim for precision vaccine development [112,119].

### 3.3. Crossing the Aisle—Heterologous Vaccines

While steering the immune response towards either mucosal or systemic immunity draws upon distinct mechanisms, the two can be combined via heterologous vaccination such as via intramuscular (IM) prime for systemic protection and intranasal (IN) pull for mucosal protection. For example, an IM prime with an mRNA-LNP vaccine followed by an IN pull with unadjuvanted spike protein generated robust mucosal and systemic immunogenicity [120]. In contrast, IM + IM or IN + IN schemes lacked effective coverage in at least one of the forms of immunity. As a proof of concept, this shows the immense potential in converging these two streams for polyfunctional immunization schemes. Similarly, while IM + IN schemes have been the key focus in heterologous schemes [121,122], future research should explore combining other routes of administration (e.g., intradermal, subcutaneous, and enteric). Considering how some of these other routes may provide more robust and/or durable protection in certain antigen/adjuvant combinations, other combinations may prove more effective.

From a precision medicine perspective, these heterologous schemes can be ideal for immunizing vulnerable populations by inducing both mucosal and systemic protection. One possible limitation of mucosal vaccines is the partly compartmentalization of the mucosal immune response from the larger systemic response [123], as the distribution of the responses depends on the actual route of induction. This separation of immune apparatuses leads to gaps in potential protection, which can be further unleased via heterologous schemes. Future studies should investigate novel heterologous combinations, including the role of adjuvantation to optimize immunogenicity and protection.

## 4. Putting It Together—Formulating Vaccine Compositions

### 4.1. Back to Basics: Alum, Polymers, and Lipids

A key aspect of vaccine development is matching an appropriate antigen and adjuvant combination with a formulation for optimal delivery and efficacy. Interestingly, the most common historical vaccine platforms (alum, polymerized formulations, and oil-in-water combinations) can be inherently immunogenic, while also enhancing the delivery efficiency of the vaccines [124,125,126]. This translates to a significant enhancement in vaccine efficacy and durability via established models that can be coupled with other novel adjuvants to boost responses, generating combination adjuvant platforms such as GSK’s adjuvantation systems [127,128].

The framework of matching antigen, adjuvant and formulation can be directly combined with precision medicine approaches to create novel combinations that enhance responses from targeted populations. As disruptive technologies and innovations such as artificial intelligence and multifactorial immune-monitoring systems elucidate insights into systemic immune responses [129,130,131], this information can help guide precision and rational vaccine design [132]. In fact, many established adjuvant-delivery systems have already been combined with novel candidates to overcome hypo-responsiveness from both early- and later-life populations [133,134,135]. These efforts are developing in parallel to existing efforts to modulate the chemo–physical properties of these canonical platforms for improved immunogenicity [125,136]. Further, the functionality of certain adjuvants is only unleashed when directly combined with appropriate immune enhancers, antigens, and/or other adjuvants. Thus, rational combinations of novel with existing systems can help integrate novel adjuvant discoveries, thereby accelerating vaccine development for vulnerable populations.

### 4.2. Strength in Numbers: Multimeric Antigenic Systems for Precision Vaccines

One of the key innovations in recent vaccine research includes advances in structure-based antigen design which can enhance immunogenicity of otherwise weak immunogens, such as fusion glycoprotein of respiratory syncytial virus [137,138,139]. Structure-based antigen optimization has been widely applied for vaccines against SARS-CoV-2, most commonly by stabilizing spike protein in trimeric prefusion conformation to improve immunogenicity [138,140]. More recently, high-density, multimeric antigens displayed onto protein or synthetic nanoparticles have demonstrated the advantage of enhancing antigen trafficking to draining lymph nodes and promoting clustering and activation of the B cell receptor, thus strengthening the magnitude and breadth of immunogenicity [141,142,143]. This is of particular importance as waning immunity is a significant concern, especially for younger children and older adults [6,144]. Thus, developing novel multimeric antigenic vaccines can be a powerful strategy to enhance and elongate protection for many vulnerable populations.

While structure-based antigen optimization is a promising approach to enhance vaccine efficacy especially in vulnerable populations, vaccines comprised of optimized antigen alone are generally insufficiently immunogenic. Clinical and preclinical studies which employed a self-assembling, two-component SARS-CoV-2 receptor-binding protein nanoparticle antigen demonstrated limited immunogenicity in the non-adjuvanted group while adjuvants such as AS03 (GSK) conferred robust protection [145,146]. Although it is possible to generalize desirable properties for an adjuvant, a proper match between an adjuvant and a specific antigen must be empirically evaluated. Evaluation of candidate antigen–adjuvant combination in pre-clinical models, such as huma in vitro and animal models, that take into account population (e.g., age, sex, and co-morbidity)-dependent vaccine immunogenicity may enable down-selecting and prioritizing adjuvanted RBD antigen-based vaccines [147].

### 4.3. Adjuvantation via mRNA Delivery Systems—Welcoming LNPs into the Club

While mRNA vaccines do not contain an adjuvant per se, they appear to be self-adjuvated. Indeed their lipid nanoparticle (LNP) delivery system not only aids in stability and delivery of the vaccine, but also stimulates innate immune responses that boost the immunogenicity of the encoded mRNA [148,149,150,151]. However, in addition to the LNP adjuvant, additional adjuvants can be utilized in mRNA-LNP vaccines to boost vaccine efficacy. From canonical adjuvants, such as cGAMP, to manganese nanoparticles, the stimulator of interferon genes (STING) pathway has been a key target to boost the innate immune response of an mRNA-LNP vaccine [152,153,154]. Further, more advanced LNP platforms containing encoding key immunological agents such as IL-12 have been used to enhance onco-immunology treatments [155,156]. These developments can be applied to enhance vaccines against infectious disease in the near future. Further, both biologic and small-molecule adjuvants can be tethered to mRNA-LNP platforms without loss of function of antigen or adjuvant recognition [157]. Thus, traditional adjuvant strategies can be powerfully combined with novel mRNA-LNP technologies to magnify quantity and quality of immune responses and extend durability of protection for vulnerable populations. Thus, as the novel mRNA-LNP technology becomes more broad ly applied to immunize against different pathogens, further adjuvantation is a practical possi bility to enhance these vaccines for populations that were less responsive to the original vaccine composition. For key components of LNPs, such as ionic lipids and pegylated polymers, rational design, including, as may be needed, optimization of these vaccine components, not just for their antigen delivery capacity but also for their inherently self-adjuvanting nature, can inform future vaccine design [128,158].

## 5. Conclusions

From discovery to development, there are multiple emerging insights and technologies in the vaccine-development space. This growing infrastructure is moving in parallel to a growing understanding of the distinct immune systems across age, sex, immune status, and co-morbidity suggesting that the traditional one-size-fits-all model of vaccine development.

**Figure 1:**
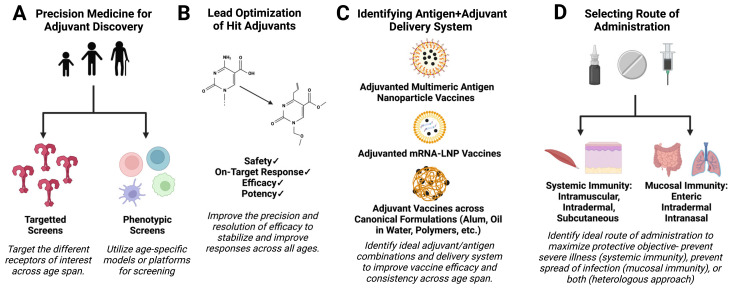
A precision-vaccinology approach to discovery and development of adjuvanted vaccines targeted to distinct vulnerable populations. Focusing discovery and innovation efforts to better understand the phenotypic and mechanistic differences in the immune systems of diverse populations, emerging research in vaccine development has explored how to optimize immunizations across the lifespan. Key elements of the precision-vaccinology approach, as applied to epidemic/pandemic response and preparedness, include (**A**) selecting robust adjuvants optimized for a target population, (**B**) modifying the chemical structure of lead adjuvants for defined functionality, (**C**) optimizing antigen and adjuvant compositions with the appropriate formulation systems, and (**D**) selecting appropriate route of administration for targeted protective correlate(s).

As illustrated (Figure 1), these insights and technologies can be combined to advance the objective of developing precision vaccines that offer highly effective protection against infectious diseases for vulnerable populations with distinct immunities.

## Figures and Tables

**Table 1:** An overview of adjuvants included in approved vaccines against infectious diseases in the United States. A table outlining adjuvants included in approved vaccines in the United States. Well-evidenced mechanisms of actions found for these adjuvants are included. Several of these adjuvants demonstrate a multifactorial mechanism of activity that is incompletely characterized, which is largely applicable to those denoted by an asterisk.

Adjuvant Name	Adjuvant Component(s)	Known Mechanism of Action
Aluminum	Salts or solutions containing aluminum	Depot effect, inflammasome, and damage-associated molecular patterns (DAMPs) activation *
AS01b/e	Monophosphoryl lipid A (MPL) and QS-21 with cholesterol liposomes	TLR4, Caspase 1, DAMPs activation *
AS03	Squalene, Tween-80, α-tocopherol	DAMPs activation *
AS04	MPL + aluminum salt	TLR4, DAMPs activation *
CpG 1018	Cytosine phosphoguanine (synthetic DNA)	TLR9 activation
Matrix-M	Saponins	DAMPs activation *
MF59	Oil in water with squalene	TLR-independent MyD88 activation *

## Data Availability

Not applicable.
